# Regression‐based machine‐learning approaches to predict task activation using resting‐state fMRI

**DOI:** 10.1002/hbm.24841

**Published:** 2019-10-22

**Authors:** Alexander D. Cohen, Ziyi Chen, Oiwi Parker Jones, Chen Niu, Yang Wang

**Affiliations:** ^1^ Department of Radiology Medical College of Wisconsin Milwaukee Wisconsin; ^2^ John Radcliffe Hospital, FMRIB Centre University of Oxford Headington Oxford; ^3^ Department of Medical Imaging First Affiliated Hospital of Xi'an Jiaotong University Xi'an Shaanxi China

**Keywords:** fMRI, machine learning, neural networks, random‐forest bootstrap aggregation, resting state

## Abstract

Resting‐state fMRI has shown the ability to predict task activation on an individual basis by using a general linear model (GLM) to map resting‐state network features to activation *z*‐scores. The question remains whether the relatively simplistic GLM is the best approach to accomplish this prediction. In this study, several regression‐based machine‐learning approaches were compared, including GLMs, feed‐forward neural networks, and random forest bootstrap aggregation (bagging). Resting‐state and task data from 350 Human Connectome Project subjects were analyzed. First, the effect of the number of training subjects on the prediction accuracy was evaluated. In addition, the prediction accuracy and Dice coefficient were compared across models. Prediction accuracy increased with the training number up to 200 subjects; however, an elbow in the prediction curve occurred around 30–40 training subjects. All models performed well with correlation matrices, which displayed correlation between actual and predicted task activation for all subjects, exhibiting a strong diagonal trend for all tasks. Overall, the neural network and random forest bagging techniques outperformed the GLM. These approaches, however, require additional computing power and processing time. These results show that, while the GLM performs well, resting‐state fMRI prediction of task activation could benefit from more complex machine learning approaches.

## 1 INTRODUCTION

Recent work has shown that resting‐state fMRI (rs‐fMRI) can be used to predict task activation on an individual basis by fitting a general linear model (GLM), where resting‐state network components are mapped to task activation (Parker Jones, Voets, Adcock, Stacey, & Jbabdi, 2017; Tavor et al., 2016). These studies work under the assumption that although underlying similarities exist between individuals’ brain responses to certain tasks, these responses differ across subjects in specific, predictable ways. For example, Tavor et al. (2016) used high spatial and temporal resolution data from the Human Connectome Project (HCP; Glasser et al., 2013; Van Essen et al., 2013) which includes four 15‐min rs‐fMRI scans and multiple tasks across several domains per subject. They extracted rs‐fMRI and structural components and mapped those components to the task activation data using a GLM. They showed intersubject variability in task activation could be predicted across several task domains including language, motor, and working memory, among others. They also were able to accurately predict language lateralization. In a follow‐up study, a GLM was used to predict task activation in a clinical population with lower spatial and temporal resolution data (Parker Jones et al., 2017). Using a covert category fluency task, they were able to accurately predict patients’ activation using a model trained on a group of healthy control subjects.

The question remains as to whether the GLM approach is optimal to accomplish this prediction. The GLM is a multiple linear regression approach and typically is used in fMRI to compute task activation. For fMRI, one or more regressors model the task, and additional nuisance regressors (i.e., motion parameters) can be added to remove unwanted signals from the data. As implied by the name, the GLM assumes a linear dependence between the variables. It also assumes the error is normally distributed, the error at each measurement is the same, and there are no correlations between the errors. These assumptions are not necessarily true for fMRI data. For the GLM prediction, the regressors are the rs‐fMRI‐derived features.

Other regression‐based learning models exist including the neural network (NN) and ensemble approaches. A typical feed‐forward NN is comprised of an input layer consisting of one or more features to be used for prediction, one or more hidden layers, and an output layer (Sperber & Karnath, 2017). Each hidden layer contains hidden nodes, or neurons, that are connected to the nodes in the subsequent layer via weighted edges. The input layer is connected to the first hidden layer and the output layer is connected to the last hidden layer. The NN machine learning approaches model nonlinearities in the data. In ensemble learning, multiple models are combined to improve learning results. One type of ensemble learning that can be used for regression is random forest bootstrap aggregation (RFbag), also referred to as “bagging.” Bagging can be used in combination with decision trees (Breiman, 2017), where data are split based on the values of the input features and terminate in leaves that contain the predicted values. In bagging, several different trees are trained on different subsets of randomly chosen data to create a so‐called forest of trees, and the values from the different trees are then averaged (Breiman, 1996, 2001). This reduces the variance. In the RFbag approach, the features are also randomly selected at each split in the decision tree.

When using the GLM approach, Tavor et al. (2016) found that not all tasks performed equally. In general, although variability existed within domains, the language, relational, and working memory domains performed better than the motor domain. Thus, one goal of this study was to evaluate whether the use of alternative approaches could improve the poorer performing domains.

In their study, Tavor et al. (2016) used only 98 subjects from the much larger HCP dataset to train and predict via a leave‐one‐out approach. Here, we have repeated the study by Tavor et al. using a larger subset of 350 HCP subjects. We examined the effect of the number of training subjects on the accuracy of the predictions. Also, we expanded on Tavor et al.'s method by comparing the NN and bagging approaches to the GLM approach. We evaluated all approaches in terms of their ability to predict task activation from rs‐fMRI based on the spatial correlation and overlap between the actual and predicted maps. Finally, we compared the spatial correlation and Dice coefficient (DC) between the approaches.

## 2 MATERIALS AND METHODS

### 2.1 Subjects

Subjects were selected from the Human Connectome Project dataset (http://www.humanconnectome.org/; Van Essen et al., 2013). In total, 350 subjects were selected for this analysis. Subjects were required to have four resting‐state scans and data from all seven task domains (described below).

### 2.2 HCP imaging

The HCP imaging protocol includes resting‐state and task fMRI data. Acquisition parameters for all datasets are described in detail in Smith et al. (2013). In short, each subject underwent four 15‐min sagittal multiband resting‐state fMRI scans with TR/TE = 720/30 ms, 2 × 2 × 2 mm isotropic resolution, and MB‐factor = 8. Scans alternated between left–right (LR) and right–left (RL) phase‐encode directions. Task fMRI data also were acquired sagittally with TR/TE = 720/30 ms, 2 × 2 × 2 mm isotropic resolution, and MB‐factor = 8. As with the resting‐state scans, scans with both LR and RL phase encoding directions were collected. Total imaging times were different based on the task but lasted approximately 5 min in general. Tasks varied across several domains including language, motor, emotion, gambling, relational, social and working memory. These are described in Barch et al. (2013). For this study, 25 contrasts across all seven domains were chosen for further analysis and are shown in Table 1. Structural and diffusion‐weighted scans were collected but were not used in this study.

**Table 1:** Prediction accuracy and statistical comparisons for the GLM, NN, and RFBag methods

		Prediction accuracy			Repeated measures ANOVA		Pairwise P‐values		
Domain	Contrast	GLM	NN	RFBag	*F*‐score	*p*‐value	NN > GLM	RFBag > GLM	RFBag > NN
Language	MATH	0.546	0.558	0.583	5,184	2.6E−87	2.6E−07	1.4E−33	8.8E−21
	STORY	0.596	0.604	0.624	3,903	2.3E−81	2.5E−04	2.5E−27	2.9E−25
MOTOR	CUE	0.673	0.689	0.702	6,572	2.5E−92	4.7E−15	1.6E−35	1.7E−13
	LF	0.528	0.536	0.557	2,569	1.3E−72	1.0E−03	8.5E−31	1.5E−15
	LH	0.507	0.521	0.544	2,298	2.5E−70	2.0E−05	3.7E−33	3.0E−15
	RF	0.531	0.542	0.559	2,957	1.5E−75	7.8E−07	2.1E−31	2.0E−15
	RH	0.509	0.517	0.545	3,049	3.5E−76	5.8E−03	6.2E−35	1.8E−17
	T	0.548	0.553	0.571	2,730	6.8E−74	2.5E−02	2.3E−28	5.4E−18
	AVG	0.536	0.546	0.565	3,247	1.7E−77	4.4E−05	1.3E−29	6.7E−15
Emotion	FACES	0.644	0.654	0.669	5,129	4.3E−87	1.5E−08	4.4E−37	1.6E−21
	SHAPES	0.624	0.631	0.655	4,420	5.8E−84	1.8E−03	6.8E−37	1.4E−25
Gambling	PUNISH	0.693	0.719	0.725	10,439	3.6E−102	7.6E−28	4.1E−40	1.7E−04
	REWARD	0.708	0.733	0.742	11,176	1.3E−103	1.4E−25	9.0E−44	2.4E−06
Relational	MATCH	0.761	0.784	0.791	11,416	4.5E−104	1.6E−27	5.7E−50	6.5E−08
	REL	0.775	0.795	0.804	14,503	3.5E−109	2.6E−21	7.7E−49	1.1E−11
Social	RANDOM	0.728	0.757	0.766	15,829	4.8E−111	3.0E−31	9.1E−46	7.2E−13
	TOM	0.759	0.786	0.794	32,426	2.2E−126	1.8E−29	8.8E−45	9.7E−12
Working memory	2BK BODY	0.680	0.698	0.706	7,575	2.4E−95	7.7E−22	9.1E−42	6.3E−09
	2BK FACE	0.662	0.685	0.693	7,504	3.8E−95	5.3E−26	3.9E−51	7.9E−07
	2BK PLACE	0.703	0.721	0.730	10,727	9.6E−103	2.2E−20	2.5E−44	3.9E−09
	2BK TOOL	0.672	0.687	0.700	5,916	4.2E−90	1.4E−15	9.1E−43	1.5E−15
	0BK BODY	0.613	0.624	0.641	3,783	1.1E−80	3.0E−09	6.5E−45	8.7E−20
	0BK FACE	0.589	0.604	0.616	5,679	3.0E−89	8.4E−12	6.1E−37	9.1E−13
	0BK PLACE	0.707	0.726	0.736	10,517	2.5E−102	5.2E−22	1.4E−42	5.4E−12
	0BK TOOL	0.683	0.694	0.710	8,194	5.2E−97	4.9E−10	1.5E−46	6.3E−18

Pairwise *p* values are Bonferroni corrected.

### 2.3 HCP preprocessing

The minimally preprocessed datasets, as described in Glasser et al. (2013) were used for all analyses. All functional data were denoised using FIX (Griffanti et al., 2014; Salimi‐Khorshidi et al., 2014). The data were then resampled to a set of 91,282 grayordinates in standard space (CIFTI; Glasser et al., 2013). This structure represents the cortex on a surface and the subcortical space and cerebellum on a voxelwise volume basis.

### 2.4 Feature extraction

All analyses were performed in MATLAB (2018). Resting‐state fMRI features were extracted following the methods outlined in Tavor et al. (2016). First, group features were derived from concatenated rs‐fMRI time series across the four scans per subject and across subjects. Group principal component analysis was performed to reduce the dimensionality to 1,000. Next, group independent component analysis was applied to each hemisphere separately to extract 40 components per hemisphere. Features with LR symmetry were kept, resulting in 34 components per hemisphere. These 34 components were then used in a dual‐regression analysis to extract individual features for all 350 subjects (Filippini et al., 2009). In the dual‐regression step, first, resting‐state data for each subject were normalized to zero mean and standard deviation equal to one. Next, each of the 34 components was used as a spatial regressor in a GLM, and the temporal signal associated with the network was extracted. This signal was then used as a regressor in a second GLM to find the spatial maps associated with each component at the individual level.

Subcortical structures were extracted as in Tavor et al. and resulted in 32 subcortical structures. To obtain the final features, the individual maps derived from dual regression and the subcortical parcellation were regressed against the individual time series to obtain one time series per component for each subject. Each time series was then correlated with each grayordinates’ time series. This resulted in 100 total features (32 subcortical plus L/R hemisphere of the 34 ICA components). The final individual feature maps (*X*_i_) were normalized to zero mean with a standard deviation equal to one and then paired with the corresponding individual *z*‐score maps derived from the task fMRI data (*y*_i_).

In addition, the brain was parcellated. Unlike the study by Tavor et al., where parcels were based on a group independent component analysis (ICA), a random parcellation scheme was used in this study with 50 parcels per hemisphere. Parcels were contiguous and were created using a Voronoi tessellation from seeds randomly spread across the surface. Parcels did not correspond to any functional or structural brain network.

Three different approaches were used to map the resting‐state features to the task data: GLM, NN, and RFBag.

### 2.5 Data preparation

**Figure 1:**
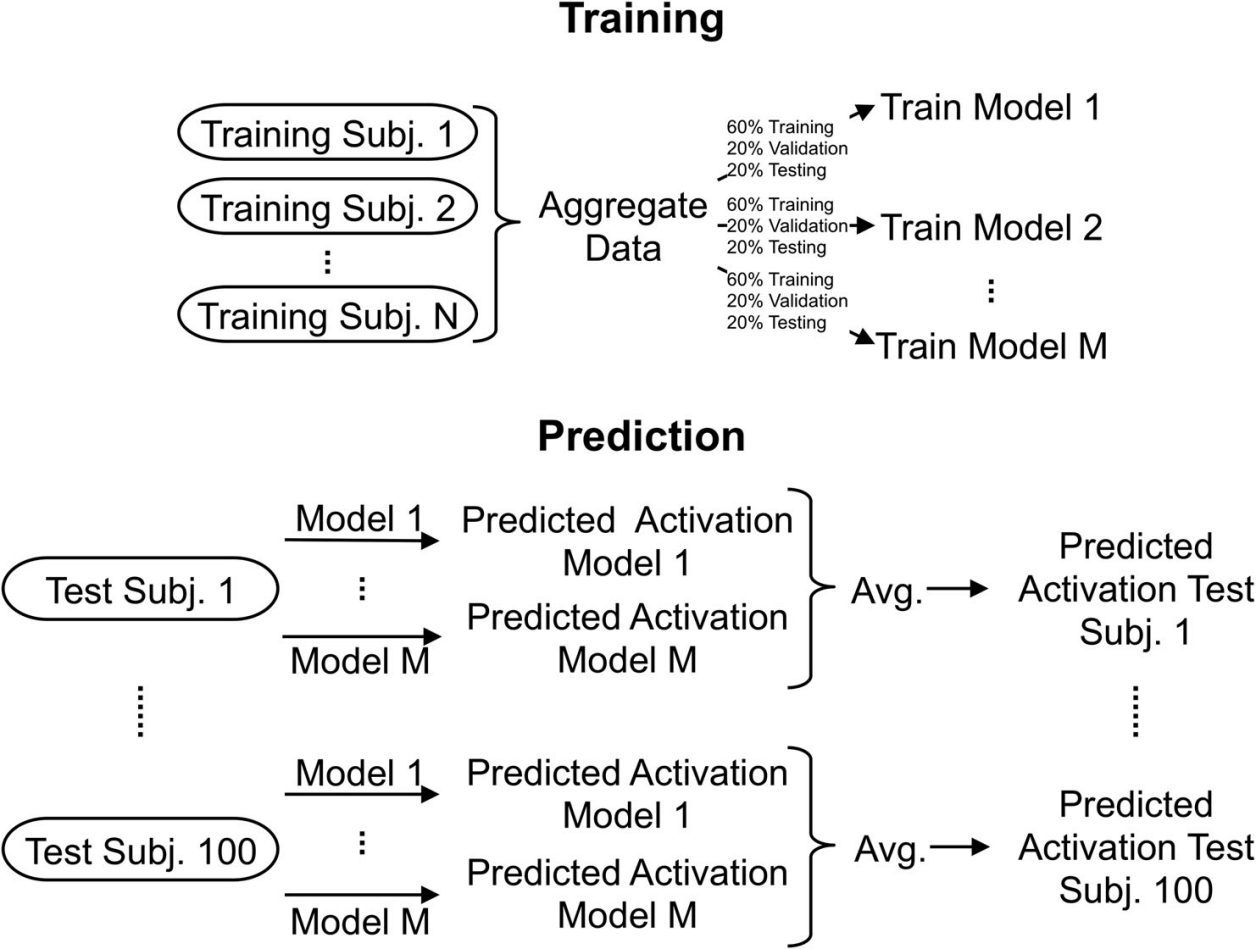
Schematic depicting the training and prediction scheme for the NN and RFBag models. In the training step, data from the N training subjects were aggregated and used to train M separate models. Data were randomly split into 60% training, 20% validation, and 20% testing for each model separately. Then, for each test subject, each of the M models was used to predict activation resulting in M predicted maps per subject. The M predicted maps were then averaged to produce one predicted map for each test subject. Both N and M were varied to examine the effects of the number of training subjects and number of averages respectively on the accuracy of the predicted maps

Subjects were randomly separated into 200 training subjects, 100 test subjects, and 50 subjects used for hyperparameter optimization for the NN and RFBag models. The same 100 test subjects were used for each model. In addition, a separate model was computed for each parcel. To examine the effect of the number of training subjects on prediction accuracy, each model was trained with 10, 20, 30, 40, 50, 100, 150, and 200 subjects selected randomly from the group of training subjects. The same training subjects were used for each model. Each of the trained models was then applied to the 100 test subjects. Training was accomplished by first, aggregating data from the training subjects to form one large dataset, which was used to train a model using the three methods. This model was then applied to the 100 test subjects. These methods are schematized in Figure 1.

### 2.6 GLM prediction

A GLM was used to determine β coefficients (β_i_) for each training subject or group of training subjects by fitting the features (*X*_*i*_) to the z‐scores (*y*_*i*_) for each task (Equation 1). A different GLM was fit for each parcel. Thus, each group of training subjects had 100 × 100 beta values, one for each feature and parcel. Trained β values (β_t_) were then matrix multiplied by each test subject's feature maps (*X*_j_) to generate predicted activation maps for that test subject (*y*_j_; Equation (2)).

(1) $$\beta_{i}=\text{pinv}\left(X_{i}\right)•y_{i}$$

(2) $$y_{j}=X_{j}∙\beta_{t}$$

### 2.7 NN prediction

**Figure 2:**
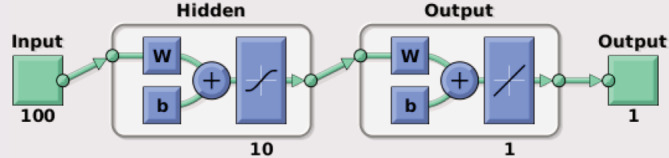
Schematic of output from the feed‐forward NN employed in this study, from the *view* command in MATLAB. In this example, the input consisted of 100 rs‐fMRI‐derived features connected to one hidden layer with 10 neurons; however, the hidden layer size varied with task according to the hyperparameter optimization. The hidden layer was, in turn, connected to the output layer, resulting in one predicted activation value for each grayordinate. W represents the weights of the connections between layers and b is the bias term

The NN approach used the same features, tasks, and parcellation scheme as the GLM approach. A feed‐forward NN was created using the *fitnet* function in MATLAB. A schematic of the NN model is shown in Figure 2.

#### 2.7.1 Hyperparameter optimization

**Figure 3:**
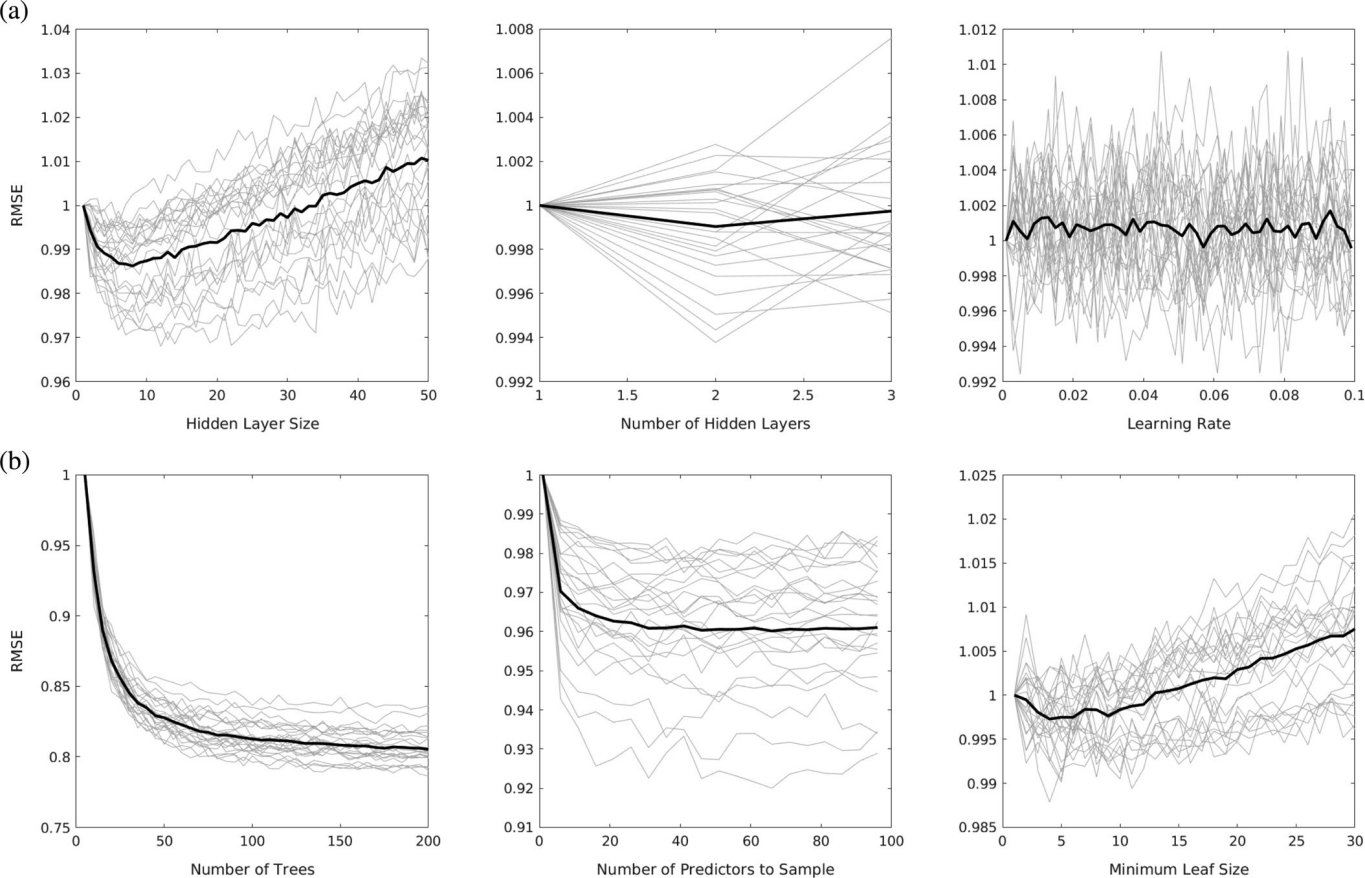
Plots of RMSE for several hyperparameters of interest for the NN model (a) and RFBag model (b). Light gray lines show individual tasks; thick black lines show the average across tasks. For comparison purposes, all plots were normalized so the first data point was equal to one

First, hyperparameter optimization was performed for each task on the 50 hyperparameter optimization subjects. For the hyperparameter optimization step, no parcellation was performed. Data was subdivided by randomly setting aside 20% of the data for testing and 20% for validation. The test data was used to evaluate model performance using the root mean square error (RMSE). Three parameters were tuned: Number of hidden layers, hidden layer size, and learning rate. First, hidden layer size was iterated from 1 to 50 and the size that minimized the RMSE was selected. Next, the number of hidden layers was iterated from 1 to 3 and the number that minimized the RMSE was selected. Finally, the learning rate was iterated from 0.001 to 0.1 at increments of 0.002. Results of the hyperparameter optimization are shown in Figure 3a. Hidden layer size had the largest effect on model accuracy. The number of hidden layers had very little impact on model accuracy. Thus, one hidden layer was chosen for subsequent analyses. Finally, RMSE fluctuated randomly with the learning rate. Therefore, a learning rate of 0.001 was chosen for subsequent analyses. The hidden layer size were varied based on task, but the same value was used regardless of the number of training subjects.

#### 2.7.2 Model training

Additional parameters for the feed‐forward NN included a tanh activation function for the hidden layer, a Nguyen–Widrow layer initialization function (Nguyen & Widrow, 1990), and the resilient backpropagation training function (Riedmiller & Braun, 1993a). The network was then trained using the *train* function, with the mean square error as the cost function. The network was train several times with the number of training subjects varying from 10 to 200 (see section 2.5). To control for overfitting, the training data was further subdivided by randomly setting aside 20% of the data for testing and 20% for validation. Training stopped when the mean square error reached a minimum on the validation data or 1,000 epochs were reached. The majority of cases reached a mean square error minimum. Trained models were then used to predict activation for the test subjects using the test subjects’ features as input.

As another means of controlling for overfitting and to improve prediction accuracy, the model was trained several times for each number of training subjects. Each trained model was used to predict activation for the test subjects and the results from each model were averaged to produce the final predicted activation map (Figure 1). Thus, for each task, 100 × #averages models were trained, one for each parcel and each average. To examine the effects of the number of averages on the prediction accuracy, 1, 5, 10, 20, and 50 averages were used for 50 training subjects.

### 2.8 Random forest bootstrap aggregation prediction

Bagging also used the same features, tasks, and parcellation scheme as the GLM approach, and a separate model was trained for each parcel. Bagging generates many decision trees by randomly selecting subsets of the data with replacement and a random subset of features at each split.

#### 2.8.1 Hyperparameter optimization

Hyperparameter optimization was also performed for the RFBag approach using the 50 hyperparameter optimization subjects and no parcellation. Data were subdivided by randomly setting aside 20% of the data for testing, which was used to evaluate model performance using the RMSE. Three parameters were tuned: Number of decision trees, number of predictors to sample, and the minimum leaf size. First, the number of trees was iterated from 1 to 200 and the size that minimized the RMSE was selected. Next, the number of predictors to sample was iterated from 1 to 99 and the number that minimized the RMSE was selected. Finally, the minimum leaf size was iterated from 1 to 30. Results of the hyperparameter optimization are shown in Figure 3b. Results were consistent across tasks. The number of trees had the largest effect on model accuracy, followed by the number of sampled predictors, and the minimum leaf size had a small effect. Based on the results, 100 trees, 33 sampled predictors, and a minimum leaf size of 5 was chosen for subsequent analyses for all tasks and all numbers of training subjects.

#### 2.8.2 Model training

Models were trained using the *TreeBagger* function in MATLAB. Trained models were then used to predict activation for the test subjects using the test subjects’ features as input. Several models were trained with the number of training subjects varying from 10–200 (see section 2.5). Also, as with the NN approach, the model was trained several times for each number of training subjects. Each trained model was used to predict activation for the test subjects and the results from each model were averaged to produce the final predicted activation map. Like the NN, for each task 100 × #averages models were trained, one for each parcel and each average. To examine the effects of the number of averages on the prediction accuracy, 1, 5, 10, 20 and 50 averages were used for 50 training subjects.

### 2.9 Model evaluation

To evaluate the prediction accuracy of each model, Pearson correlation coefficients (CC) were calculated between each test subject's predicted maps and all other test subjects’ task maps, resulting in a 100 × 100 matrix for each approach and task. For these matrices, a strong diagonal component indicates actual and predicted activation maps are more similar for the same subject compared with the other subjects, and therefore predict individual activation well. The matrices were row and column normalized to account for the different variances of the actual and predicted maps. The correlation between actual and predicted activation was compared across models with a repeated‐measures analysis of variance (ANOVA). Maps were also thresholded for each subject and modeled separately using a mixture model consisting of a Gaussian and two gamma functions (Jones et al., 2017), and the DC was computed (Equation (3)). The median of the upper gamma was used as the threshold. The DC was also compared across models using repeated‐measures ANOVA. Finally, to examine the effect of the number of training subjects and number of averages on accuracy, the mean CC value between actual and predicted activation was plotted versus number of training subjects and number of averages respectively for each approach and task.

It is important to note there were two methods used for model performance evaluation. During training, model performance was evaluated using the mean square error on the testing subset of data from the training subjects. Once the models were trained, they were used to predict activation for the 100 separate test subjects. Here prediction accuracy was defined as the Pearson's correlation between the actual and predicted tasks.

(3) $$DC=\frac{2\left|A_{1}\cap A_{2}\right|}{\left|A_{1}\right|+\left|A_{2}\right|}$$

## 3 RESULTS

### 3.1 Effect of the number of training subjects

**Figure 4:**
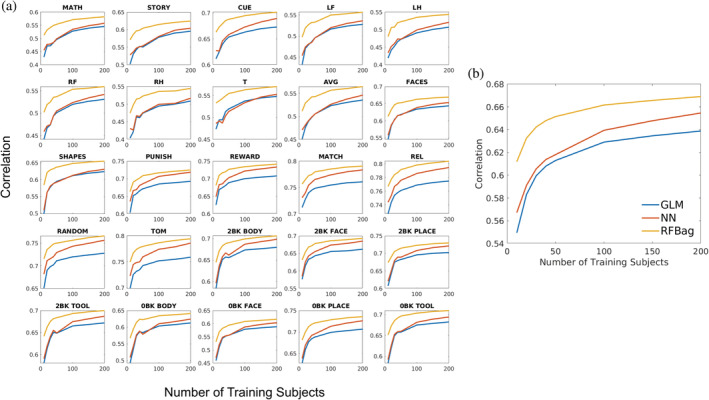
Plots showing the correlation between actual and predicted activation as a function of the number of subjects used for training for all approaches and all 25 tasks (a) and averaged across tasks (b). The plots for all approaches and the majority of tasks have an elbow around 30 or 40 training subjects, although the correlation continues to increase up to 200 subjects. Also, for all of tasks, the RFBag approach had the highest correlation across training numbers

Figure 4 shows plots of the mean CC values as a function of the number of training subjects for all approaches and tasks. For the majority of approaches and tasks, the plots of the CC values had an elbow around 30–40 subjects; however, increases in CC were seen up to 200 subjects. The results discussed in this section are all reported for 200 training subjects.

### 3.2 Effect of the number of averages

**Figure 5:**
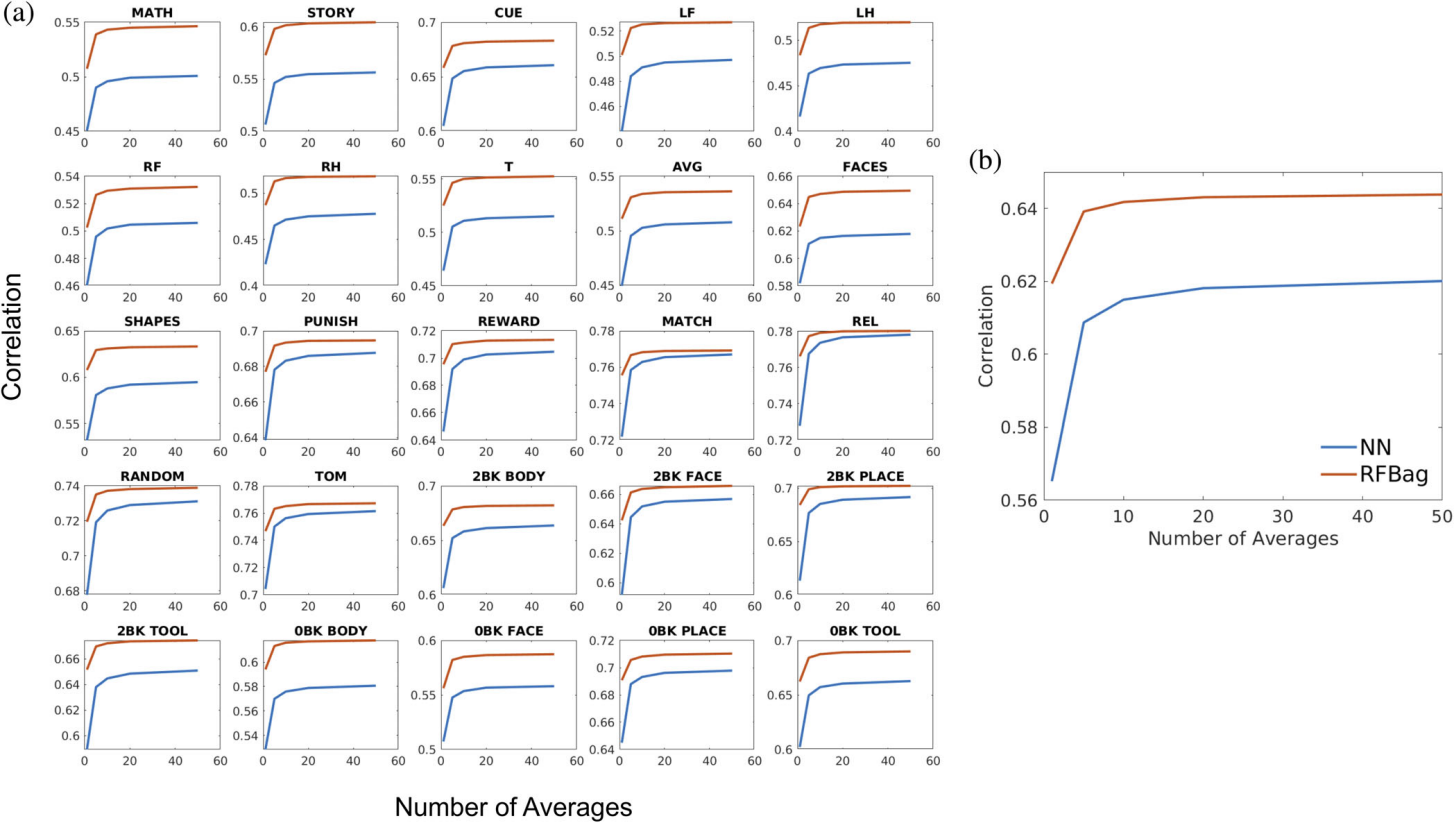
Plots showing the correlation between actual and predicted activation as a function of the number of models trained and averaged for all approaches and all 25 tasks (a) and averaged across tasks (b). For the NN model, the correlation reached an asymptote around 20 averages. The RFBag model, the correlation did not significantly past five averages. Averaging multiple models did not have as large of an effect for the RFBag model compared to the NN model, although correlation was higher for the RFBag model across averages. In some cases, NN correlation with 50 averages did not reach the RFBag correlation with no averages

Figure 5 shows plots of the mean CC values as a function of the number of averages for the NN and RFBag approaches and all tasks. For the NN approach, the plots of the CC values had an elbow around 20 averages. For the RFBag approach, an elbow was seen around five averages. Thus, 20 averages were used for the NN approach and five averages were used for the RFBag approach.

### 3.3 Comparison to Tavor et al.

First, we were able to replicate the results of Tavor et al. in a set of 350 HCP subjects, which included 252 subjects not included in the original study. We saw strong diagonalization of the normalized and nonnormalized CC matrices across tasks using the GLM approach.

### 3.4 Prediction accuracy (correlation)

Overall, as in Tavor et al. (2016) and Jones et al. (2017), both the actual and predicted activation maps varied between subjects in terms of the strength and size of activation. For all approaches, prediction accuracy varied across the task domain and between tasks within the same domain. For example, for the motor domain, mean CC between actual and predicted activation ranged from 0.544 for the LH task to 0.702 for the CUE task for the RFBag approach. In general, the gambling, relational, and social domains performed the best, with a mean CC > 0.7, while the motor domain performed the worst, with a mean CC < 0.6. Mean CC values for all methods and tasks are summarized in Table 1. Quantitatively, the RFBag and NN approach outperformed the GLM approach, with significantly higher CC values for all tasks. In addition, the RFbag approach had significantly higher CC compared to the NN approach for all tasks.

**Figure 6:**
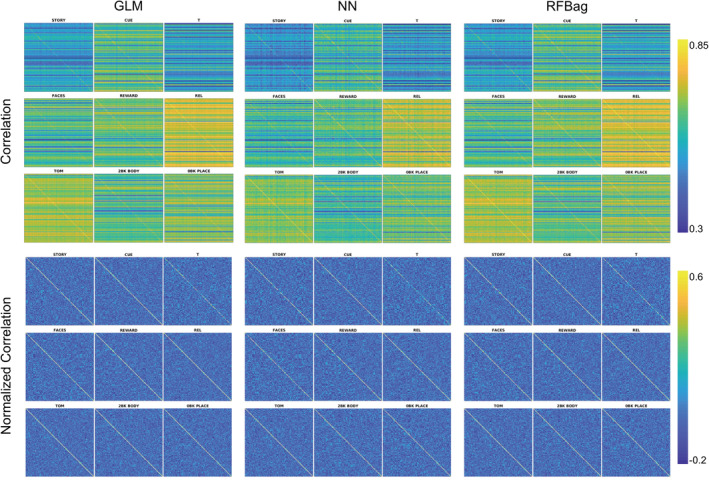
Non‐normalized (top) and normalized (bottom) correlation matrices for the GLM (left), NN (middle), and RFBag (right) approaches. Nine tasks are shown, including at least one task from each of the seven domains. Higher correlation along the diagonal can be seen for all approaches and tasks. The diagonal trend is strongest for REL and TOM tasks. The diagonal is heightened for the normalized correlation matrices. Qualitatively, the matrices look very similar across approaches, although the diagonal is slightly more prominent for the RFBag approach

Correlation maps between actual and predicted activation were constructed and show the correlation between the actual activation map and the predicted activation maps of all other subjects. They showed a higher correlation along the diagonal for all approaches and tasks, indicating the predicted activation maps more closely matched the actual maps for the same subject compared to all other subjects. This diagonal trend was stronger for the gambling, relational, and social domains compared with the others. Correlation maps were also row and column normalized to allow for comparison across subjects. The diagonal component was heightened for the normalized matrices. The normalized and non‐normalized CC matrices were qualitatively similar across approaches. Example matrices from nine tasks are shown in Figure 6.

### 3.5 Dice coefficient

**Figure 7:**
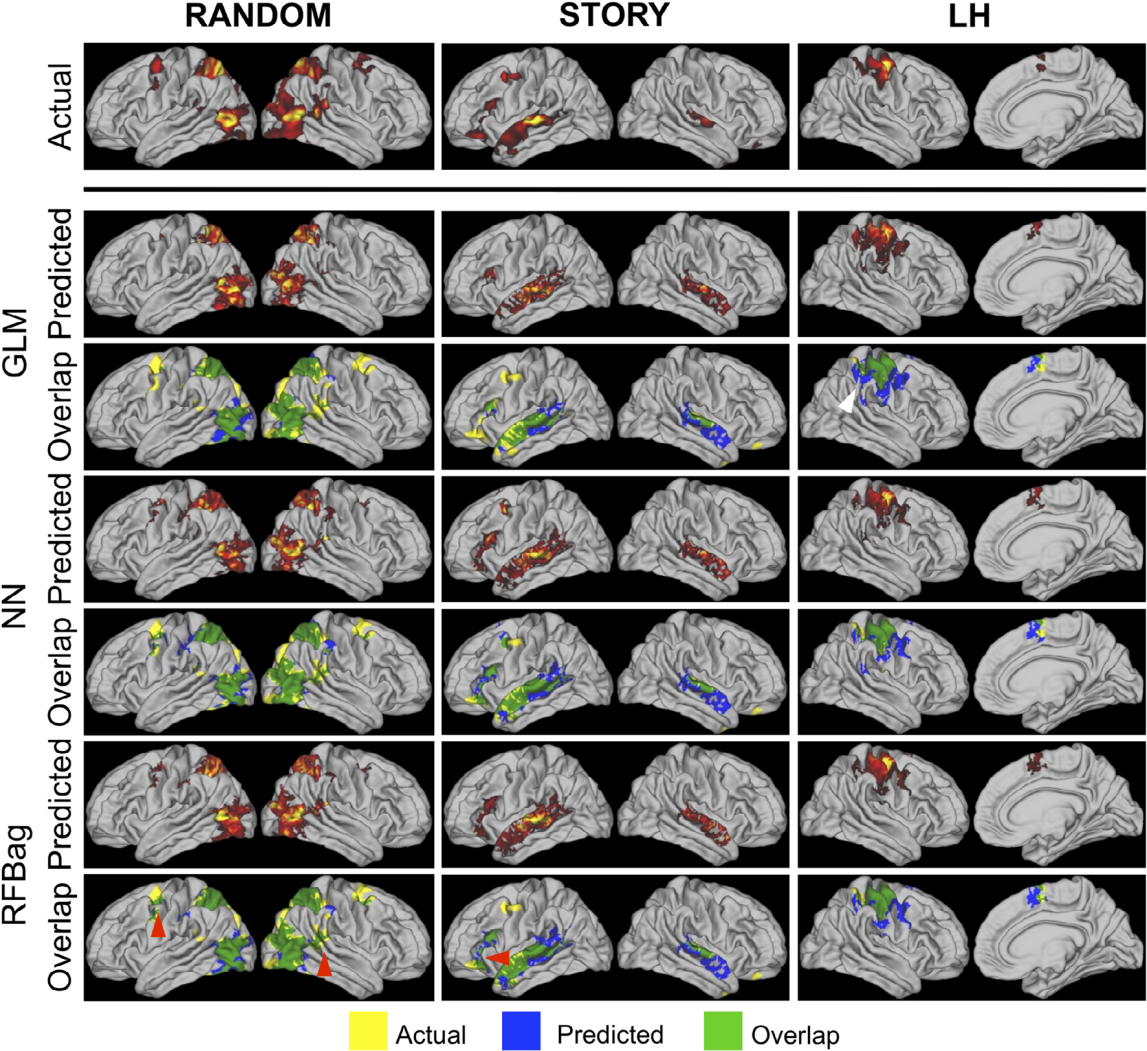
Example actual (top), predicted (middle), and overlap (bottom) maps for the GLM, NN, and RFBag models for the RANDOM (left), STORY (middle), and LH (right) tasks for one representative subject. Only positive activation is shown. Significant overlap is seen for all three approaches and tasks; however, additional overlap can be seen for the RFBag approaches as illustrated by the red arrows. For the LH case, the GLM model approach resulted in more spurious predicted activation compared to the NN and RFBag approaches (white arrow)

Maps were thresholded using a mixture model method and used to calculate the DC. Overall, the DC results mirrored the CC results. The emotion, gambling, relational, and social tasks performed the best with DC > 0.5 for all tasks within those domains. The RFBag approach performed the best, with a significantly higher DC for the majority of tasks as compared with the other approaches. Quantitative DC results are shown in Table 2. Visually, the overlap was similar across approaches. Figure 7 shows examples of actual, predicted, and overlap maps for the RANDOM, STORY, and LH tasks.

**Table 2:** Dice coefficient and statistical comparisons for the GLM, NN, and RFBag methods

		Dice coefficient			Repeated measures ANOVA		Pairwise *p*‐values		
Domain	Contrast	GLM	NN	RFBag	*F*‐score	*p*‐value	NN > GLM	RFBag > GLM	RFBag > NN
Language	Math	0.308	0.316	0.328	952	1.3E−52	1.7E−01	3.0E−05	2.1E−02
	Story	0.494	0.508	0.514	1,581	1.1E−62	1.6E−02	7.0E−05	1.8E−01
Motor	CUE	0.108	0.202	0.138	271	4.3E−30	2.3E−15	1.6E−04	3.1E−11^a^
	LF	0.321	0.327	0.333	873	6.6E−51	4.7E−02	2.0E−08	7.4E−02
	LH	0.284	0.295	0.297	600	8.4E−44	3.7E−02	1.7E−02	1.0E+00
	RF	0.311	0.317	0.320	841	3.5E−50	2.4E−02	1.6E−05	3.2E−01
	RH	0.280	0.286	0.291	699	1.2E−46	1.1E−01	4.5E−05	2.5E−01
	T	0.324	0.336	0.345	725	2.4E−47	1.8E−05	2.0E−12	1.5E−03
	AVG	0.297	0.302	0.309	927	4.5E−52	3.8E−01	1.1E−06	2.3E−02
Emotion	Faces	0.466	0.472	0.480	856	1.6E−50	9.6E−02	2.7E−08	5.5E−05
	Shapes	0.379	0.387	0.391	486	5.7E−40	2.3E−02	1.2E−03	3.1E−01
Gambling	Punish	0.450	0.477	0.480	1,516	7.8E−62	8.6E−09	3.7E−10	8.7E−01
	Reward	0.477	0.499	0.505	2,200	2.0E−69	4.9E−10	1.8E−13	5.8E−02
Relational	Match	0.467	0.500	0.512	2,005	1.6E−67	1.0E−19	2.2E−31	7.1E−08
	REL	0.484	0.497	0.509	1,966	4.0E−67	4.3E−05	2.1E−12	3.7E−05
Social	Random	0.485	0.516	0.520	1,614	4.3E−63	7.1E−14	9.3E−18	7.1E−02
	TOM	0.469	0.493	0.496	1,846	7.9E−66	9.1E−11	2.7E−13	3.1E−01
Working memory	2BK Body	0.453	0.453	0.468	2,975	1.1E−75	1.0E+00	2.5E−11	2.9E−08
	2BK Face	0.433	0.445	0.451	2,502	4.4E−72	5.4E−04	1.5E−09	2.9E−02
	2BK Place	0.497	0.507	0.451	3,242	1.8E−77	1.4E−03	6.3E−19	2.6E−09
	2BK Tool	0.442	0.452	0.462	2,640	3.4E−73	1.9E−04	7.2E−16	4.6E−04
	0BK Body	0.437	0.441	0.457	1,715	2.5E−64	3.4E−01	6.6E−13	5.4E−11
	0BK Face	0.421	0.434	0.440	1,748	1.0E−64	4.9E−04	6.0E−08	7.2E−02
	0BK Place	0.519	0.545	0.556	2,032	8.6E−68	3.4E−09	2.8E−15	1.4E−04
	0BK Tool	0.497	0.509	0.523	2,087	2.4E−68	8.4E−05	5.8E−16	9.2E−07

a NN > RFBag; Pairwise *p* values are Bonferroni corrected.

## 4 DISCUSSION AND CONCLUSIONS

In this study, several regression models to predict task activation from rs‐fMRI were compared, including GLMs, neural networks, and random forests. First, the effect of the number of training subjects on accuracy was analyzed by varying the number of subjects used for training from 10 to 200 subjects. Accuracy increased with training number for all approaches up to 200 subjects, but an elbow occurred around 30–40 subjects. The effect of averaging the results of multiple models was also investigated for the NN and RFBag approaches. Increasing accuracy was seen as a function of number of averages. In addition, the results from Tavor et al. (2016) in which a GLM was used, were replicated on a set of 350 HCP subjects. Similar results were found in our analysis, including a strong diagonalization of the cross‐correlation matrices and variable correlation strengths across tasks. Next, the analysis was expanded using more complex regression models. Significantly higher accuracy, defined as the correlation between actual and predicted activation, was achieved by using the RFBag approach compared to NN and GLM approaches.

The effect of the number of training subjects on prediction accuracy was analyzed for all approaches and tasks. CC increased with training number for all tasks and models up to the maximum number of subjects (200) tested. These results indicate all models can benefit from additional training subjects; however, practically, 200 subjects may not be feasible. These results also indicate similar results can be obtained for the GLM approach with 200 subjects and the NN and RFBag approaches with 30–50 subjects. In addition, although the CC versus training number curves increased up to 200 subjects, they have an elbow at 30–40 raining subjects, which indicates that fewer training subjects may produce adequate results.

The accuracy of both the NN and RFBag approaches improved by averaging the results of several trained models; however, the NN approach benefitted more. This averaging technique essentially creates an ensemble method, which acts as a regularizer decreasing bias and/or reducing variance. The random forest method is already an ensemble method, averaging the predictions from multiple models. Thus, averaging the results of several models had a smaller effect and tended to level off around five averages. On the other hand, the NN technique is not inherently an ensemble method, so more averages had a larger effect.

There are several NN training types available in MATLAB, including resilient backpropagation, Levenberg–Marquardt, and scaled conjugate gradient backpropagation, among others. Of the methods we tested, we found that the resilient back propagation method worked best for the type of analysis used in this study. Resilient back propagation is a gradient descent algorithm that is dependent on only the sign, not the magnitude, of the partial derivatives (Riedmiller & Braun, 1993a). This approach typically converges faster than other gradient descent algorithms.

One issue with NN approaches is that they have the potential to overfit the training data, which leads to a model that fits the training data very well but does not generalize when applied to new data. To account for this, 20% of the training data was set aside as test data and 20% as validation data. Throughout the training, the model was tested on the test data, and the loss function (i.e., mean square error) was computed on the validation data. The training stopped when the validation loss function was minimized. The test data is not seen by the model when training, which allows the model to be tested on unseen data. In this way, the generalizability of the model can be maintained. Another approach to reduce NN overfitting is the dropout method where nodes and their connections are randomly dropped during training (Srivastava, Hinton, Krizhevsky, Sutskever, & Salakhutdinov, 2014). This creates a number of “thinned” networks. During testing, all nodes remain and their weights are multiplied by the probability a node is present at training time. It may be worthwhile comparing this method to the NN approach with averaging as it also creates a NN ensemble.

Although hyperparameter optimization was used for the RFbag approach, another option that can be used is feature reduction, which determines the importance of individual features and excludes less important features from the training process. Feature reduction was not used in this study.

The HCP dataset consists of more than 80 task contrasts collected across seven task‐fMRI scans. Previous work by Tavor et al. analyzed 43 of these contrasts and found a wide variability in the ability of rs‐fMRI to predict task activation, with correlation values between actual and predicted task activation ranging from 0.12 for the GAMBLING−PUNISH−REWARD task to 0.80 for the RELATIONAL−MATCH task (Tavor et al., 2016). In this study, 25 tasks were selected to provide a range of correlation values to evaluate the ability of the more advanced regression approaches to improve predictions for both “good” and “bad” tasks across several task domains. Thus, several motor tasks were chosen that performed poorly in the Tavor et al. study. These included MOTOR−LH, MOTOR−RH, MOTOR−LF, and MOTOR−RF. We also selected tasks that performed well in the Tavor et al. study. These include gambling, relational, social, and multiple WM‐2BK tasks. For all tasks, the NN and RFbag models resulted in higher actual and predicted task correlation compared with the GLM, and they did not improve the poorer performing tasks more than the better‐performing tasks.

It does appear, however, that the NN prediction accuracy was closer to the RFBag prediction accuracy for tasks with higher correlation in general. For example, the majority of motor tasks had mean correlation values <0.6 (Table 1 and Figure 4). For these tasks, there was a large gap between the NN and RFBag correlation values. For the PUNISH and REWARD tasks where correlation values were greater than 0.7, the NN and RFBag correlation values were much closer. For many of the tasks with lower correlation values, there was not much difference between the GLM and NN approaches, especially with lower numbers of training subjects.

Despite significantly higher CCs between actual and predicted maps for the RFBag and NN models compared with the GLM, both the un‐normalized and normalized CC matrices (Figure 6) looked qualitatively similar, and the diagonal component was strong for the majority of tasks for all approaches. Furthermore, overlap maps of actual and predicted activation also appeared qualitatively similar despite the significantly higher DC for the NN and RFBag models compared with the GLM. Of note, the DC and the overlap maps themselves are dependent on the threshold chosen. Here, a mixture model was used to threshold the data by fitting a Gaussian and two gamma functions—one positive and one negative—to the histogram of *z*‐scores. This was done separately for the actual task, predicted task, model, and subject, and considered the lower values on average for the predicted compared with the actual task *z*‐scores.

The GLM is significantly faster and uses fewer computational resources compared with the NN and bagging approaches. For example, the NN model was trained using a GPU on a Linux workstation with 256 GB of RAM and a GeForce GTX 1080 Titan graphics card. Training all subjects took approximately 30 s per subject for the NN model, whereas it took <1 s for the GLM, and prediction for the individual approaches took up to 2 min per subject when the number of training subjects was 200. Thus, if time is limited and/or the necessary computational resources are unavailable, the GLM is a viable option.

This study was not without limitations. First, all approaches were trained on a voxelwise (or gray ordinate wise) basis for each parcel. Thus, aside from the parcellation, neither model incorporated spatial information. Including a spatial component to the model could further improve results. Machine learning approaches such as convolutional neural networks (CNNs) inherently take spatial information into account. CNNs can take 2D or 3D images as input to train a model. They have been used in MRI for image reconstruction, tissue segmentation, tumor segmentation, and fMRI analysis (Choi & Jin, 2016; Jang, Plis, Calhoun, & Lee, 2017; Kamnitsas et al., 2017; Meszlenyi, Buza, & Vidnyanszky, 2017; Pham, Ducournau, Fablet, & Rousseau, 2017; Qin et al., 2019; Valverde et al., 2017). CNNs may be worth considering in future studies. This study used HCP data, which has high temporal and spatial resolution, and 1 hr of rs‐fMRI scans per subject. Clinical data typically are of lower quality and have shorter scan times and lower resolution. Furthermore, patient data tend to be more variable compared with that of control subjects. Parker Jones et al. (2017) found promising results by extending the GLM model to a group of patients who performed a category fluency task with only 5 min of resting‐state fMRI (TR = 3.5 s). Future studies are underway to apply these models in a patient setting. As mentioned, feature selection was not employed in this study. More extensive hyperparameter tuning and feature selection might be explored in future studies.

In conclusion, advanced regression techniques were used to predict task activation from rs‐fMRI data. All models accurately predicted task activation for a wide range of task domains on an individual basis; however, higher correlation between actual and predicted task activation was seen for the RFBag model compared to the NN model and the previously studied GLM.

## Data Availability

The data that support the findings of this study are available from the corresponding author upon reasonable request.
